# The Modifications of Haemoglobin, Erythropoietin Values and Running Performance While Training at Mountain vs. Hilltop vs. Seaside

**DOI:** 10.3390/ijerph18189486

**Published:** 2021-09-08

**Authors:** Maria Cristina Man, Cătălin Ganera, Gabriel Dan Bărbuleț, Michał Krzysztofik, Adelina Elena Panaet, Alina Ionela Cucui, Dragoș Ioan Tohănean, Dan Iulian Alexe

**Affiliations:** 1 Department of Physical Education, 1 Decembrie 1918 University of Alba Iulia, 510009 Alba Iulia, Romania; cristina.man@uab.ro (M.C.M.); gabriel.barbulet@gmail.com (G.D.B.); 2 Nicolae Rotaru Sports Program High School of Constanţa, 900178 Constanța, Romania; cataganera@yahoo.com; 3 Institute of Sport Sciences, The Jerzy Kukuczka Academy of Physical Education in Katowice, 40-065 Katowice, Poland; m.krzysztofik@awf.katowice.pl; 4 Doctoral School, National University of Physical Education and Sport Bucharest, 060057 Bucharest, Romania; adelina_panaet@yahoo.com; 5 Department of Physical Education and Sports, Valahia University of Targoviste, 130024 Targoviste, Romania; haralambiealina2008@yahoo.com; 6 Faculty of Physical Education and Mountain Sports, Transilvania University of Brasov, 500036 Brasov, Romania; 7 Faculty of Movement, Sports and Health Sciences, VasileAlecsandri University of Bacău, 600115 Bacău, Romania; alexedaniulian@ub.ro

**Keywords:** altitude, haemoglobin, erythropoietin, hypoxia, endurance, sand

## Abstract

Altitude training increases haemoglobin, erythropoietin values among athletes, but may have negative physiological consequences. An alternative, although less explored, that has the potential to positively influence performance while avoiding some of the negative physiological consequences of hypoxia is sand training. Ten endurance-trained athletes (age: 20.8 ± 1.4, body mass: 57.7 ± 8.2 kg, stature: 176 ± 6 cm; 5000 m 14:55.00 ± 0:30 min) performed three 21-day training camps at different locations: at a high altitude (HIGH), at the sea-level (CTRL), at the sea-level on the sand (SAND). Differences in erythropoietin (EPO) and haemoglobin (Hb) concentration, body weight, VO_2max_ and maximal aerobic velocity (VMA) before and after each training cycle were compared. Data analysis has indicated that training during HIGH elicited a greater increase in VO_2max_ (2.4 ± 0.2%; *p* = 0.005 and 1.0 ± 0.2%; *p* < 0.001) and VMA (2.4 ± 0.2%, *p* < 0.001 and 1.2 ± 0.2%; *p* = 0.001) compared with CTRL and SAND. While increases in VO_2max_ and VMA following SAND were greater (1.3 ± 0.1%; *p* < 0.001 and 1.2 ± 0.1%; *p* < 0.001) than those observed after CTRL. Moreover, EPO increased to a greater extent following HIGH (25.3 ± 2.7%) compared with SAND (11.7 ± 1.6%, *p* = 0.008) and CTRL (0.1 ± 0.3%, *p* < 0.001) with a greater increase (*p* < 0.01) following SAND compared with CTRL. Furthermore, HIGH and SAND elicited a greater increase (4.9 ± 0.9%; *p* = 0.001 and 3.3 ± 1.1%; *p* = 0.035) in Hb compared with CTRL. There was no difference in Hb changes observed between HIGH and SAND (*p* = 1.0). Finally, athletes lost 2.1 ± 0.4% (*p* = 0.001) more weight following HIGH vs. CTRL, while there were no differences in weight changes between HIGH vs. SAND (*p* = 0.742) and SAND vs. CTRL (*p* = 0.719). High-altitude training and sea-level training on sand resulted in significant improvements in EPO, Hb, VMA, and VO_2max_ that exceeded changes in such parameters following traditional sea-level training. While high-altitude training elicited greater relative increases in EPO, VMA, and VO_2max_, sand training resulted in comparable increases in Hb and may prevent hypoxia-induced weight loss.

## 1. Introduction

High-altitude training has long been considered to improve the performance of endurance athletes [1]. As the altitude increases, the quantity of oxygen decreases, resulting in adaptations to the conditions of reduced oxygenation [2]. These changes include an increased oxygen-carrying capacity (i.e., increased haemoglobin mass) among other non-haematological changes [2]. Meta-analyses data suggests that the performance of elite endurance athletes can improve by ~4–5% following methods of live high-train low and live high-train high altitude training [3]. However, some athletes may not respond favourably to such practices potentially due to the negative physiological consequences of training in a hypoxic environment (e.g., impaired sleep quality, weight loss, decrease in muscle protein synthesis) [4,5]. 

An alternative, although less explored, practice that has the potential to positively influence performance while avoiding some of the negative physiological consequences of hypoxia is sand training [6,7]. Evidence suggests that the unique training adaptations for sand may have a positive influence on endurance performance [8,9]. Sand surfaces may provide a training stimulus that elicits a higher energetic cost with less ground reaction force compared to the more traditional training surfaces, such as synthetic fabrics or grass [10]. The high shock absorption capacity of sand may decrease the impact forces experienced during high-intensity activities, which may lead to a reduction in muscle lesions and pain and improve recovery time between sessions [10,11]. Considering these differences, recent evidence supports the use of sand as a training means to improve the performance of team sports athletes [12]. Binnie et al. [7] have reported that for training, the use of sand instead of grass training surfaces elicits a relatively higher training intensity, without causing any additional decrement in performance the following day (24 h post exercise). Furthermore, in a study by Yiǧit and Tuncel [13], a significant improvement in predicted VO_2max_ over the 6-weeks was reported in the sand running group but not in the road running group. Therefore, sand has the potential of providing not only a unique training stimulus for athletes but also a viable option for recovery sessions.

Recently, more complex research exploring specific mechanisms that may lead to greater adaptations with sand versus more traditional training surfaces has emerged [7,12,13,14]. Such studies have quantified the contributions of energetic uptake (aerobic and anaerobic) during short-term exercising in the sand (namely 10 min) [14]. A study by Binnie et al. [7] investigated the effect of sand surfaces during a training session comprising running at different speeds for a longer period (60 min). Specifically, these studies compared the use of sand and grass surfaces during training sessions [7,9,11,15]. For a session functioning with standardised intervals, the use of sand instead of grass or synthetic surfaces led to a significantly higher average heart rate (sand: 172 bpm; grass: 163 bpm) and blood lactate values (sand: 10.1 mmol ·L^−1^, grass: 6.5 mmol · L^−1^) throughout the training session [13,16].

Given that the energy consumption of sand running is higher compared to training on traditional training surfaces, such as grass, this may lead to greater physiological adaptation over a specific training period. Therefore, we sought to compare differences in haemoglobin (Hb), erythropoietin (EPO) concentrations and running performance (maximal aerobic velocity [VMA] and capacity [VO_2max_]) in highly trained runners following 21-day high-altitude, sea-level/sand, and traditional sea-level training cycles. We hypothesized that high-altitude and sea-level training on sand will significantly increase blood parameters and physical performance in comparison to the sea-level training, while a slightly greater improvement will be noted after high-altitude training.

## 2. Materials and Methods

### 2.1. Participants and Study Design

Participants were: 10 male athletes (age: 20.8 ± 1.40, body mass: 57.7 ± 8.2 kg, stature: 176 ± 6 cm), who specialised in middle-distance and long-distance events, mountain running, with 5000 m 14:55.00 ±0:30” at the Romanian National Championships and at International Championships. The main inclusion criteria were: a compete in the national-level event and that the participants were free from musculoskeletal injuries for at least 6 months before each training cycle of the study. The athletes went through the same training program for 21 days across three training cycles separated by one year. Standard meals were provided to the athletes at each training camp. Additionally, all athletes adhered to the same regimen of vitamin and mineral supplementation. Anthropometric measures, blood draws, and running performance tests were taken on the day before and the first day after each 21-day training program to determine changes in body weight, haemoglobin concentration, and running performance. Blood samples were taken following a 12 h fast at the same time of day (±1 h) at each timepoint. Samples were stored for <48 h at 2–8 °C prior to analyses. A timeline of assessments taken before and after each 21-day training program is provided in Table 1.

### 2.2. Training Programs

The athletes covered three training periods, as follows: In the first stage, the athletes carried out a training cycle for 21 days at Piatra Arsă, at an altitude of ~2000 m. In the second stage, the same group of athletes carried out the same training routine at the seaside, on the Black Sea coast, at Mamaia-Constanța (~0 m). Finally, the third training cycle took place in Blaj, at an altitude of ~600 m.

The timing and geographical locations of each training cycle were:

- 1 August 2017–22 August 2017, National Sports Complex “Piatra Arsă” of the Bucegi Mountains (altitude; HIGH G1)
- 1 August 2018–22 August 2018, on the Black Sea coast, in Mamaia-Constanța (sea-level/SAND; G2)
- 1 August 2019–22 August 2019, in Blaj, the Alba County (traditional sea-level, 600 m CTRL)

A sample training program from the “Piatra Arsă” cycle is detailed in Table 2. Similar training programs in terms of effort zones were used during the training periods on the Black Sea Coast and in the locality of Blaj, with the exception that within the training period at the seaside the accommodation period was reduced from 7 days (at high altitude) to 3 days and during the research stage at Blaj there was no accommodation period.

### 2.3. Anthropometrics

The bodyweight of participants was determined in the morning, using the same digital scale (ZET27288; SC Zetman Kraft SRL, Arad, Romania) and recorded to the nearest 100 g. 

### 2.4. Haemoglobin Concentration

Hb was determined from venous blood collected before and after each training program using a Diagon D-Cell 60 automated haematology analyser (Diagon Ltd., Budapest, Hungary).

### 2.5. Erythropoietin Concentration

The serum concentration of EPO was determined by the commercially available Human EPO Quantikine™ IVD ELISA Kit (R&D Systems, Minneapolis, MN, USA).

### 2.6. Maximal Aerobic Velocity and Capacity

The five-minute test consisted of the athletes running at full capacity for five minutes. VMA = distance obtained [in km] × 12. The five-minute test was used to determine VMA and the formula VMA × 3.5 was used to predict VO_2max_ because all subjects were over 18 years of age [17]. The previous study confirms it as a reliable and practical indirect method to estimate individual aerobic fitness in a trained population [18].

Chamoux et al., 1996 [19,20] mention that the VMA determined on the terrain depends on the duration of the effort and therefore on the protocol used. Examining the relationship between running speed and running time log established from world racing records foot shows a significant point at 4.97 min, proposed as the reference time for VMA. By convention, VMA could be measured on the field by a 5 min test regardless of the sport.

### 2.7. Statistical Analyses

Statistical analyses were performed using SPSS software (v.26, IBM, Armonk, NY, USA). Repeated measures ANOVA was used to determine whether characteristics (e.g., body weight, [Hb], [EPO], VMA, VO_2max_) remained similar prior to each 21-day training camp. Changes in these characteristics across each training camp were detected using one-way ANOVA. Repeated measures ANOVA were further used to determine differences in the relative change (% difference from pre-training) in characteristics across training camps. A Bonferroni correction was used for multiple pairwise comparisons and when sphericity was violated, the Greenhouse–Geisser corrected was used. Normality was assessed using Shapiro–Wilk’s test. Statistical significance was set a priori at *p* < 0.05. 

## 3. Results

### 3.1. Sample Size

The sample size analysis was performed using G*Power software (Dusseldorf, Germany). Given the study 2-way analysis of variance (ANOVA) (three conditions and two repeated measures), a moderate overall effect size (ES) = 0.58, an alpha-error < 0.05, the desired power (1-ß error) = 0.8, and correlation among repeated measures = 0.5, the total sample size resulted in nine participants. This value of effect size was chosen according to the improvements in VO_2max_ after training on the sand from Binnie et al. [7].

### 3.2. Anthropometrics 

The body weight of the athletes was similar prior to each 21-day training camp (*p* = 0.133). Body weight decreased by 1.3 ± 0.6 kg (*p* < 0.001), from 57.7 ± 8.2 kg before the training period to 56.4 ± 8.0 kg after HIGH. There was no change in body weight from before the training period to after SAND (*p* = 0.149; pre = 57.8 ± 7.9 kg vs. post = 57.0 ± 7.8 kg) or from before the training period to after CTRL (*p* = 0.504; pre = 59.4 ± 8.3 kg vs. post = 59.3 ± 8.1 kg). 

There tended to be an overall difference (*p* = 0.59, ƞ_p_^2^ = 0.318, 1-β = 0.507) in the percentage of body weight change from pre- to post-training between training cycles. Athletes lost 2.1 ± 0.4% (*p* = 0.001) more weight following HIGH vs. CTRL, while there were no differences in weight changes between HIGH vs. SAND (*p* = 0.742) and SAND vs. CTRL (*p* = 0.719). 

### 3.3. Maximal Aerobic Velocity and VO_2max_

VO_2max_ was higher (*p* = 0.015) before the HIGH (66.7 ± 0.6 mL/kg/min) vs. CTRL (63.3 ± 0.8 mL/kg/min) training period. VO_2max_ before SAND (65.4 ± 0.6 mL/kg/min) was not different from values before HIGH (*p* = 0.142) or CTRL (*p* = 0.231). Similarly, MAV was higher (*p* = 0.015) before HIGH (19.06 ± 0.18 km/h) vs. CTRL (18.10 ± 0.23 km/h). MAV before SAND (18.70 ± 0.18 km/h) was also not different from HIGH (*p* = 0.096) or CTRL (*p* = 0.135).

VO_2max_ increased by 1.7 ± 0.4 mL/kg/min following G1 (*p* < 0.001) and by 1.0 ± 0.2 mL/kg/min following G2 (*p* < 0.001). VMA increased by 0.48 ± 0.10 km/h following G1 (*p* < 0.001) and by 0.24 km/h following G2 (*p* < 0.001). However, training during G3 did not elicit a change in VO_2max_ (*p* = 0.146) or VMA (*p* = 0.452).

**Figure 1:**
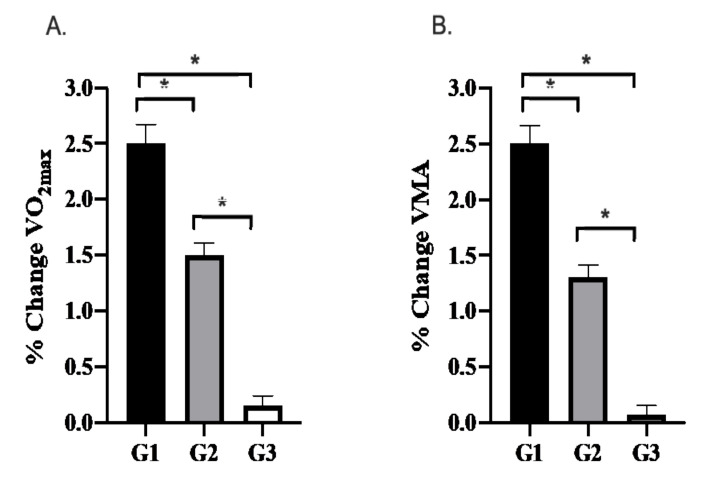
The relative change (pre-post) in (**A**) VO_2max_ and (**B**) VMA following 21-days of training at altitude (G1), seaside (G2), and sea-level (G3). Values displayed are the means ± SD. Significant differences (*p* < 0.05) between training camps are denoted as (*).

There was an overall effect of training cycle on the relative change in VO_2max_ (*p* < 0.001, ƞ_p_^2^ = 0.896, 1-β = 1.0) and VMA (*p* < 0.001, ƞ_p_^2^ = 0.969, 1-β–1.0). Training during G1 elicited a 2.4 ± 0.2% greater increase in VO_2max_ (*p* = 0.005) and VMA (*p* < 0.001) compared with G3. Training during G1 also elicited a 1.0 ± 0.2% greater increase in VO2max (*p* < 0.001) and a 1.2 ± 0.2% greater increase in VMA (*p* = 0.001) compared with G2. Increases in VO_2max_ following G2 were 1.3 ± 0.1% greater (*p* < 0.001) and in VMA were 1.2 ± 0.1 (*p* < 0.001) than those observed after G3. Figure 1 shows the relative change in performance data following each 21-day training camp.

### 3.4. Erythropoietin Concentrations

EPO values differed prior to the start of each training camp (*p* = 0.003) with lower values noted before the start of G1 (5.5 ± 2.1 mU/mL) compared to G2 (8.2 ± 0.6 mU/mL; *p* = 0.03) and G3 (9.0 ± 0.5 mU/mL; *p* = 0.009). Increases in EPO were elicited only with G1 (*p* < 0.001; post = 6.9 ± 2.8 mU/mL) and G2 (*p* < 0.001; post = 9.2 ± 1.9 mU/mL) training cycles. Whereas EPO were unchanged after G3 (*p* = 0.678, post = 9.0 ± 1.7 mU/mL). 

Training cycle had a significant effect on the relative change in EPO (*p* < 0.001, ƞ_p_^2^ = 0.829, 1-β = 1.0). EPO increased to a greater extent following G1 (25.3 ± 2.7%) compared with G2 (11.7 ± 1.6%, p = 0.008) and G3 (0.1 ± 0.3%, *p* < 0.001). Further, the relative increase in EPO was greater (*p* < 0.01) following G2 compared with G3. 

### 3.5. Haemoglobin Concentrations 

**Figure 2:**
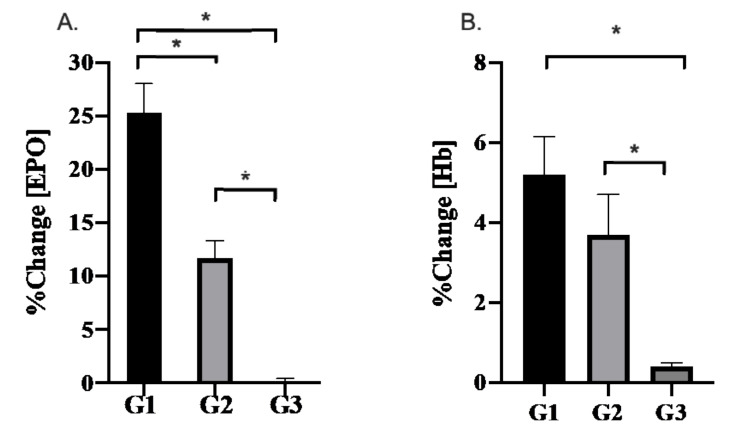
The relative change (pre-post) in (**A**) [EPO] and (**B**) [Hb] following 21-days of training at altitude (G1), seaside (G2), and sea-level (G3). Values displayed are the means ± SD. Significant differences (*p* < 0.05) between training camps are denoted as (*).

Hb measured before the start of each training camp were similar (*p* = 0.145). Each training camp provoked increases in Hb with a 0.8 ± 0.4 g/dL increase observed after G1 (*p* < 0.001; pre = 14.4 ± 0.9 g/dL vs. post = 15.2 ± 1.0 g/dL), a 0.5 ± 0.4 g/dL increase after G2 (*p* < 0.001; pre = 13.8 ± 1.1 g/dL vs. post = 14.3 ± 0.8 g/dL), and a 0.1 ± 0 g/dL increase after G3 (*p* = 0.003). Training cycle had a significant effect on the relative change in Hb (*p* < 0.001, ƞ_p_^2^ = 0.884, 1-β = 1.0). G1 elicited a 4.9 ± 0.9% greater increase (*p* = 0.001) in Hb compared with G3. G2 elicited a 3.3 ± 1.1% greater increase (*p* = 0.035) in Hb compared with G3. There was no difference in Hb changes observed between G1 and G2 (*p* = 1.0). Figure 2 displays the relative change in EPO and Hb following each training period. 

## 4. Discussion

This study aimed to evaluate the changes in EPO, Hb concentrations and running performance (VMA and VO_2max_) following 21-day high-altitude, on the sand at sea level, and traditional sea-level training cycle. Our primary findings are that both, high-altitude training and sea-level training on sand resulted in significant improvements in EPO, Hb, VMA, and VO_2max_ that exceeded changes in such parameters following traditional sea-level training. While high-altitude training elicited greater relative increases in EPO, VMA, and VO_2max_, sand training resulted in comparable increases in Hb and may prevent hypoxia-induced weight loss. The main conclusions of this study confirmed our initial hypothesis and suggest that the physiological modifications triggered by 21-days of either high-altitude training or training on the sand at sea level are greater than those attained by traditional sea-level training methods.

The effects of altitude training have been widely examined [21,22,23,24,25,26], however, there is scarce research on the training on the sand [7,9,15]. Training on soft sand led to a significant increase in heart rate (HR> 22 bpm), oxygen uptake rate (VO_2_ > 0.872 L·min^−1^) compared to a hard surface, thus clearly demonstrating a higher level of physiological effects experiences while exercising on sand compared to traditional surfaces [10]. Moreover, when comparing the training on the sand and on the grass, similar levels of haemolysis (serum haptoglobin concentration) were reported when they are followed by physical exercise. [27]. Exercise-induced haemolysis, the moment when the red blood cells are destroyed, is characterised by the free increase in haemoglobin and by a decrease in blood haptoglobin provides an alternative method of measuring the degree of stress on the musculoskeletal system during exercise [28] or competitive stress [29]. Foot problems are a major cause of haemolysis, and this appears during running sessions. The variables which are involved in training sessions such as the type of terrain and the experience can influence the quantity of haemolysis. Consequently, training on sand may lead to lower levels of haemolysis suffered during exercise, due to the decrease in impact forces experienced when touching the ground. Alternatively, haemolysis may increase with a higher frequency of the contacts with the ground; it has also been proven to increase with the intensity of exercise possibly due to the compression of capillary networks, by activating lean mass to a higher degree or due to higher tissue hypoxia levels, leading to RBC oxidative stress [30]. Furthermore, the increase in EPO and haemoglobin values after training at the seaside can be the consequence of several factors: running on the sand [9,15] negative ions [31] present in large quantities on the seashore, amounts similar to altitude, wind (sea breeze) that can cause hypoxia. Moreover, training on sand leads to an increased alteration of kinematic running (cadence, joint angles, etc), muscle activation patterns [32] and physiological responses [16].

The numerous studies on the effects of training in hypoxia show that one of the most important effects of altitude training concerns the increase in haemoglobin mass [33,34,35,36,37,38]. The mechanism of improvement seems to stem from an increase in EPO driven by stabilization of its transcription factor hypoxic inducible factor-α in the low PO_2_ environment [33]. As such, a greater O_2_ carrying capacity with chronic exposure to hypoxic environments with altitude training practices have been cited to contribute to improved submaximal and maximal exercise capacity [34]. For example, it has been shown—in a study conducted for 27 days at 2500 m—that the concentration of haemoglobin increases regularly, due to EPO concentration, featuring a 100% increase on the first day of stimulation, while the concentration of soluble receptors recorded 19% after only 19 days [39]. Wehrlin and Marti, 2006 [40] have shown—upon analysing two world-class runners in 5000 m and marathon—increases of 3.9 and 7.6% in haemoglobin mass and 5.8 and 6.3% in erythrocytes after a live high-train low training camp that lasted 28 days, 18 h/day, at an altitude of 2456 m, with training at 1800 m. This finding is interesting and practical because it shows a positive effect among world-class athletes for whom inducing even a slight gain in fitness level is a challenge. In a different study, the same authors [40] have studied well-trained triathletes and a control group: they have concluded, also using the live-high and train-low method for the altitude of 2500 m, an 18 h/day and training in 1800 and 1000 m, an increase in haemoglobin mass by 5.3% and of red cells by 5.0%, as well as improving sports performance by increasing VO_2max_ values by 4.1%. Comparing this study with the one we conducted, we see similar changes. At 2000 m altitude, after 21 days of preparation, haemoglobin increased by 4.9% and VO_2max_ values by 2.4%.

Although most studies indicate favourable physiological and physical changes in increasing athletes’ performance, altitude training also has negative effects such as a decrease in body weight (in the present study by 2.1%) [41,42,43,44]. However, no such differences were observed in the current study after training on the sand. Therefore, this training approach induces a comparable increase in Hb concentration to altitude training and may prevent hypoxia-induced weight loss. Nevertheless, it must be mentioned that high-altitude training elicited greater relative increases in EPO, VMA, and VO_2max_.

Considering the training studies carried out thus far, additional research is necessary to determine a whole array of benefits associated with training on the sand. Namely, the main characteristic associated with exercising on sand is higher movement energy cost [11,15] and the capacity of reaching higher training intensities during a training session is higher compared to training on a traditional surface [45,46]. Consequently, additional research is required to investigate the implications of training on the sand, with a focus on aerobic and muscular adaptations and the causes that trigger changes in the concentration of EPO and haemoglobin.

Our study is not without limitations. Although the study was conducted on elite runners, the sample size was small. Moreover, we have measured Hb concentration rather than Hb mass. Furthermore, the EPO concentrations were significantly lower prior to the high-altitude training compared with other training—this could have possibly led to the greater increases seen in this parameter following training. In addition, the differences in weather—the higher ambient temperatures on the seaside—could have led to changes in Hb mass (w/heat training).

## 5. Conclusions

The results of this study suggest that the physiological modifications triggered by 21-days of either high-altitude training or training on sand at sea-level are greater than those attained by traditional sea-level training methods. However, it must be mentioned that high-altitude training elicited greater relative increases in EPO, VMA, and VO_2max_, while sand training resulted in comparable increases in Hb and may prevent hypoxia-induced weight loss. These findings are of practical value as they suggest that training on sand at sea-level can be considered as an alternate for high-altitude training. In this regard, when the introduction of high-altitude training is impossible, the coaches and athletes could incorporate training on sand at sea-level.

## Figures and Tables

**Table 1:** Graph of evaluations.

Blood Sampling		Anthropometric Measurements		Physical Tests to Determine VO_2max_/VMA	
Before 21-day training program	After 21-day training program	Before 21-day training program	After 21-day training program	Before 21-day training program	After 21-day training program
31 July 2017 8:00 o’clock	22August2017 8:00 o’clock	31 July 2017 8:30 o’clock	22 August 2017 8:30 o’clock	31 July 2017 16:00 o’clock	22 August 2017 16:00 o’clock
31 July 2018 8:00 o’clock	22 August 2018 8:00 o’clock	31 July 2018 8:30 o’clock	22 August 2018 8:30 o’clock	31 July 2018 16:00 o’clock	22 August 2018 16:00 o’clock
31 July 2019 8:00 o’clock	22 August 2019 8:00 o’clock	31 July 2019 8:30 o’clock	22 August 2019 8:30 o’clock	31 July 2019 16:00 o’clock	22 August 2019 16:00 o’clock

VMA = maximum aerobic velocity. VO_2max_ **=** maximal aerobic capacity.

**Table 2:** Training program: Piatra Arsă (2000 m).

Day	Piatra Arsă-2000 m Altitude	Total Km- Running
**1**	**T.S._1_** 8 km e.r. 50%VMA/**T.S._2_** 8 km e.r. segment strength-65%VMA	**16**
**2**	**T.S._3_** 10 km e.r. 50%VMA/**T.S._4_** 6 km e.r. and 3 complete strength series-65% VMA	**16**
**3**	**T.S._5_** 12 km r. uniform tempo, 65%VMA, segm. strength/**T.S._6_** 10 km r. uniform tempo, 10 × 100 m a.l.-65% VMA	**22**
**4**	**S.T._7_** 16 km r. various land, segment strength and r.l.-70%VMA	**16**
**5**	**S.T._8_** 20 km r. various land-75% VMA/**S.T._9_**- 8 km e.r. 3 series of ex. for strength	**28**
**6**	**S.T._10_** 16 km r. uniform tempo. 70%VMA/**S.T._11_** 10 km r. uniform tempo., 10 × 100 m r.l.-70% VMA	**27**
**7**	**S.T._12_** 14 km r. uniform tempo., 75%VMA	**14**
**8**	**S.T._13_** 16 km r. progressive various land 75–83% VMA/**S.T._14_** 10 km r. uniform tempo., -70% VMA and 3 series of ex. for strength	**26**
**9**	**S.T._15_** 6 km r. uniform tempo.,65%VMA, 20 × 100 m r. accelerated (100%VMA) with connection 100 m e.r. 4 km/**S.T._16_** 10 km r. uniform tempo., stretching	**24**
**10**	**S.T._17_** 10 km e.r., stretching 75%VMA/**S.T._18_** 40 min r. (2 min r. tempo sustained + 1 min conn.+1 min r tempo sustained +1 min. connection) x 8 series (90%VMA)	**26**
**11**	**S.T._19_** 15 km r. various land (75–80%VMA)	**15**
**12**	**S.T._20_** 10 km r. various land and 10 × 100 m r.l. with 100 m e.r. 80%VMA/**S.T._21_** 10 km r. uniform tempo., (75%VMA)	**24**
**13**	**S.T._22_** 12 km r. tempo. progressive-88–93% VMA/**S.T._23_** 10 km e.r. (75%VMA)	**26**
**14**	**S.T._24_** 16 km r. various land 80% VMA/**S.T._25_** 10 km r. uniform tempo., (75%VMA)	**26**
**15**	**S.T._26_** 14 km r. various tempo 92–94%VMA, 1 km e.r.	**27**
**16**	**S.T._27_** 10 km e.r. 75%VMA/**S.T._28_** 14 km e.r. segment strength (60%VMA)	**24**
**17**	**S.T.**_29_ 26 km r. various land (65%VMA)	**26**
**18**	**S.T._30_** 6 km e.r. 15 × 100 m with 100 m (95%VMA), 3 km e.r./**S.T._31_** 10 km r. uniform tempo, 70%VMA	**22**
**19**	**S.T._32_** 8 km e.r.75%VMA/**S.T._33_** 3 km e.r. 20 × 300 m with connection 100 m e.r. (30 sec) 100%	**20**
**20**	**S.T._34_** 2 hours’ walk- forest (2000 m/600 m altitude)/beach (0 m altitude)	**0**
**21**	**S.T._35_** 15 km r. 80%VMA/**S.T._36_** 10 km r. uniform tempo., 75%VMA	**25**

r. = running; e.r. = easy running; r.l. = running launched; S.T._1_ = training session 1; VMA = maximum aerobic velocity.

## Data Availability

The datasets used and analysed during the current study are available from the corresponding author on reasonable request.
